# Early-Life Exposure to the Chinese Great Famine and Later Cardiovascular Diseases

**DOI:** 10.3389/ijph.2021.603859

**Published:** 2021-03-09

**Authors:** Zhenghe Wang, Yanhui Dong, Rongbin Xu, Xijie Wang, Yanhui Li, Zhiyong Zou

**Affiliations:** ^1^ Department of Epidemiology, School of Public Health, Southern Medical University, Guangzhou, China; ^2^ School of Public Health and Institute of Child and Adolescent Health, Peking University, Beijing, China; ^3^ Department of Epidemiology and Preventive Medicine, School of Public Health and Preventive Medicine, Monash University, Melbourne, Vic, Australia

**Keywords:** cardiac epidemiology, fetal medicine, community child health, public health, child nutrition

## Abstract

**Objectives:** This study aimed to examine the association between early life famine exposure and adulthood cardiovascular diseases (CVDs) risk.

**Methods:** A total of 5,504 subjects were selected using their birthdate from national baseline data of the China Health and Retirement Longitudinal Survey to analyze the association between famine exposure in early life and CVDs risk in adulthood. CVDs was defined based on the self-reported doctor’s diagnosis.

**Results:** The prevalence of CVDs in the unexposed group, fetal-exposed, infant-exposed, and preschool-exposed groups was 15.0%, 18.0%, 21.0%, and 18.3%, respectively. Compared with the unexposed group, fetal-exposed, infant-exposed and preschool-exposed groups had higher CVDs risk in adulthood (*p* < 0.05). Compared with the age-matched control group, infancy exposed to famine had a significantly higher adulthood CVDs risk (OR = 1.52, 95% CI: 1.15, 2.01; *p* = 0.006). The association seems to be stronger among population with higher education level (*P*_interaction_ = 0.043). Sensitivity analysis revealed consistent association between early-life famine exposure and adult CVDs risk.

**Conclusion:** Early life exposed to the China great famine may elevate the risk of CVDs in adulthood.

## Introduction

Cardiovascular diseases (CVDs) is the leading cause of mortality both in high-income countries and developing countries [1]. In China, cardiovascular deaths has accounted for 45% and 43% of total deaths in rural and urban areas, respectively [2]. The burden of CVDs is increasing and has become a major public health problem affecting 290 million people in China [3]. The emerging epidemic of CVDs in China was explained by urbanization, lifestyle changes, and the accelerated population aging, but now increasing attention is being paid to severe early-life malnutrition of the generation who born during “1959–1961 China Great famine” [4].

From the spring of 1959 to the fall of 1961, China experienced three years of extreme food shortages. This event is known as the “Three-year natural disaster” or “China’s Great famine”, which affected 600 million Chinese, resulted in approximately thirty million premature deaths, and the same number of births were lost or postponed, most of them died from hunger-related causes [5]. The survivors born during that period have reached their late 50s.

Developmental Origins of Health and disease (DOHaD) hypothesis, since reported firstly by professor David Barker in 1986, has become well accepted among scientists and researchers in medical and biological sciences [6]. Briefly, the hypothesis speculated that malnutrition in early life increased the risk of various non-communicable diseases in later life [7, 8]. Despite evidences from animal models suggested that poor nutrition condition in early life elevated the risk of adverse health outcome [9], direct evidence in human is still limited due to ethnical limitations. Existing studies in human mainly came from several natural history famine birth cohorts, such as Dutch Winter famine [10], 1959–1961 China Great famine [11].

In the past few decades, a rapid increasing number of studies have observed that early-life exposed to the 1959–1961 China Great famine is associated with the elevated risk of hypertension [12], diabetes [13], metabolic syndrome [14], dyslipidemia [15], chronic lung disease [16], and arthritis in later life [17]. These associations were further supported by our recently epigenetic findings, which showed that early-life exposure to the 1959–1961 China Great famine is associated with higher methylation level in the *INSR* and *IGF2* genes and higher total cholesterol [18, 19].

However, the association between famine exposure in early life and risk of CVDs in later life remains unclear. In the Dutch famine cohort, Roseboom et al showed that the association of famine exposure with coronary heart disease risk was stronger in early gestation than that in mid or late gestation [20]. However, results from a cohort of the siege of Leningrad did not observe a direct effect of early-life famine on the prevalence of CVD [21]. Moreover, Ekamper et al did not found a significant association between prenatal famine exposure and CVD risk [22]. Several studies from the Chinese famine in 1959–1961 suggested that early-life famine exposure could strengthen the association between hyperglycemia, hypertension, diabetes, hyperglycaemia and CVDs [23–25], however, these studies did not examine the association of the Chinese famine exposure with CVD risk. Moreover, Du et al observed that early-life famine exposure elevated the risk of CVD in later life [26]. The Chinese famine occurred between 1959 and 1961, but Du et al defined participants who born between January 1, 1959 and December 31, 1962 as fetal exposed group. Thus, some participants might be not exposed to famine in their fetal period, which might underestimate the effect of famine exposure on CVDs.

In this context, the current study aims to explore the association between exposure to famine in several early life stages (prenatal, infant, and preschool) and CVDs risk in adulthood using data from the baseline survey of the China Health and Retirement Longitudinal Survey (CHARLS).

## Methods

### Participants

**FIGURE 1:**
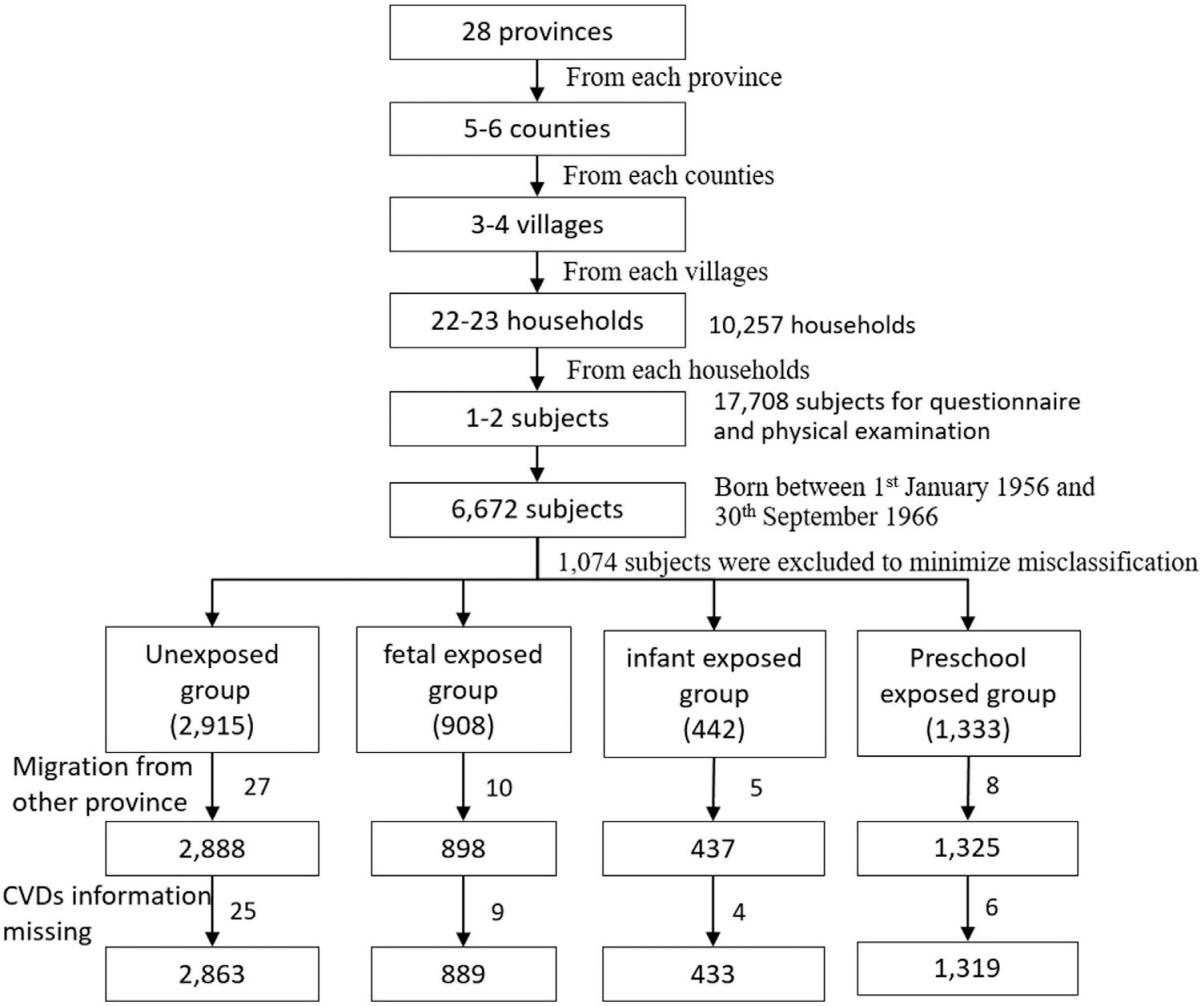
The Flowchart on the sample selecting methods at each step (China, 2011–2012).

All the participants were selected from national baseline survey of the CHARLS program. It is a large national epidemiological program performed by the National School of Development in Peking University. The program focused on the health and retirement of middle and older adults aged ≥45 years. The detailed protocol had published elsewhere [27]. Briefly, the national baseline data were collected using the face-to-face household interview by trained interviewers in 28 provinces in mainland China from June 2011 to March 2012. The multi-stages cluster random sampling method was used to select the subjects from 10,257 households, 450 villages/neighborhoods, 150 counties, 28 provinces. Finally, 17,708 subjects were recruited in the baseline survey. Health status and function information was collected using a structured questionnaire. In the current study, we firstly selected 6,672 subjects born between January 1, 1956 and September 30, 1966. To minimize misclassification, we excluded 439 subjects born from January 1, 1959 to September 30, 1959, and 635 subjects born from October 1, 1961 to September 30, 1962. Furthermore, after excluding 50 subjects with migrating from another province to the current province of residence and 44 subjects without CVDs information, 5,504 subjects with complete self-reported CVDs information were selected in the final analysis (Figure 1). In addition, we excluded 1,636 subjects born between October 1, 1962 and September 30, 1964 from unexposed group to perform the sensitivity analysis.

### Ethics Approval Statement

The current study is a secondary analysis of the CHARLS public data, which have been approved by the Medical Ethics Committee of Peking University (IRB00001052–11015).

### Definitions of Famine Exposure Groups and Severity

**FIGURE 2:**
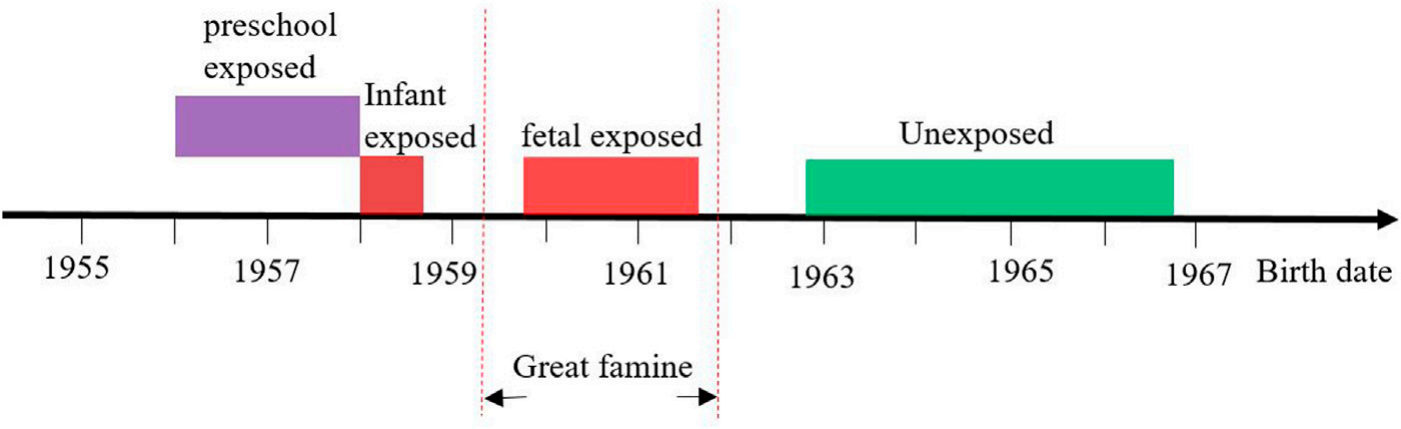
The definition of different famine exposure group according to date of birth (China, 2011–2012).

Considering the China Great famine lasted for three years from the spring of 1959 to the fall of 1961, and almost affected all Chinese lived in mainland China, the current study used birthdate of subjects to define famine exposed groups and unexposed groups (Figure 2). Preschool-exposed group (01/Jan/1956–31/Dec/1957), infant-exposed group (01/Jan/1958–30/Sep/1958), fetal-exposed group (1/Oct/1959–30/Sep/1961), and unexposed group 1 (1/Oct/1962–30/Sep/1966).

Although the China Great famine affected almost the entire region of mainland China, the severity of famine exposure varied across provinces due to different climate, agriculture policies and grain distribution system during famine period. Same as the previous study, [28], the excess mortality was used to reflect the severity of famine exposure due to lack of objective indicators such as daily calorie intake. The excess mortality was calculated using the formula listed below:

$$\text{Excess}\;\text{mortality}=\frac{\text{Average}\;\text{mortality}\;\text{in}\;1959\;\text{to}\;1961-\text{Average}\;\text{mortality}\;\text{in}\;1956-1958}{\text{Average}\;\text{mortality}\;\text{in}\;1956-1958}\times 100\%.$$

The range of excess mortality across China mainland was great, from 11.4% in Tianjin to 474.9% in Anhui province. The 50.0% was used as a threshold to categorize all the region into severe affected areas (excess mortality ≥50.0%) and less severe affected areas (excess mortality <50.0%).

### Definition of Cardiovascular Diseases

CVDs was defined based on self-reported information in the current study. Subjects were defined as CVDs if he/she had been diagnosed with stroke or cardiac diseases (including heart attack, coronary heart disease, angina, congestive heart failure, or other heart problems, such as chest pains when climbing stairs/uphill or walking quickly) by a doctor. Previous studies have showed that self-reported CVDs is relatively reliable [29, 30].

### Covariates

General demographic, socioeconomic and lifestyle factors was collected using the structured national baseline questionnaire according to a standard procedure. The highest education attainments of subjects and their parents were categorized into four groups: primary school and below, junior school, senior school, college and above. The international physical activity questionnaire short form (IPAQ-SF) [31] was used to assess the physical activity (PA) level of and all subjects were categorized into light PA, moderate/vigorous PA groups. According to the alcohol use information, all the subjects were classified into former/current drinker (subjects who ever or current drank at least one-unit alcohol per month), and never drinker (subjects who never consumed alcohol in their life). According to smoking status, all the subjects were categorized into former/current smoker (subjects who ever or current smoked at least one cigarette in the past year), and never smoker (subjects who smoked less than 100 cigarettes in lifetime). Body mass index (BMI) was calculated using weight (kg) divided by height square (m^2^), and subjects were defined as overweight/obese (if BMI ≥24.0 kg/m^2^) and normal weight (if BMI <24.0 kg/m^2^).

### Statistical Analyses

The chi-square test and ANOVA were used to compare the difference between famine exposed groups and unexposed group of categorical variables and continuous variables, respectively. Logistic regression model was used to analyze the association between famine exposure in early life and CVDs risk in adulthood. Model 1, unadjusted for any covariate. Model 2, adjusted for gender, Model three further adjusted for smoking status, drinking status, physical activity level, education level, parent’s education level, BMI status, and famine severity.

To minimize the effect of age difference on the association between famine exposure and risk of CVDs, we combined the unexposed and preschool exposed groups as age-matched control group, and used the age-matched control group as a reference group to analyze the association between fetal and infant exposed groups and CVDs risk in adulthood.

In addition, we also performed a sensitivity analysis after excluding subjects born between October 1st, 1962 and September 30th, 1964 from unexposed group.

All analyses were performed by SPSS 20.0 (IBM Corporation, Armonk, NY, USA). Statistical significance was considered to be when *p* < 0.05 (two sided).

### Patient and Public Involvement

The patients and the public were not involved in the design of this study, in the selection of the outcomes, in the conduct of the study or in result dissemination.

## Results

Basic characteristics are presented in Table 1. A total of 5,504 subjects with complete self-reported CVDs information were enrolled in the current study. The prevalence of CVDs in the unexposed group, fetal-exposed, infant-exposed, and preschool-exposed group was 15.0%, 18.0%, 21.0%, and 18.3%, respectively. Compared with the unexposed group, fetal-exposed, infant-exposed and preschool exposed groups had higher prevalence of CVDs (*p* = 0.001). The mean age of three famine-exposed groups was older than the unexposed group (*p* < 0.001). Compared with the unexposed group, infant-exposed and preschool-exposed groups had lighter weight (*p* < 0.05), and preschool-exposed group had smaller BMI (*p* = 0.006). In addition, distributions of gender, smoking status and education level of subjects were significant different among four groups (*p* < 0.05). However, we did not observe any difference for height, physical activity level, drinking status, and overweight/obese among four groups (*p* > 0.05).

**TABLE 1:** Basic characteristic of study population according to the China famine exposure (China, 2011–2012).

	Unexposed group	Fetal exposed group	Infant exposed group	Preschool exposed group	*p*
Birth date	10/1/1962–9/30/1966	10/1/1959–9/30/1961	1/1/1958–12/31/1958	1/1/1956–12/31/1957	
N	2,863	889	433	1,319	
Female, n (%)	1,564 (54.7)	467 (52.6)	210 (48.5)	658 (50.0)	0.008
CVDs, n (%)	430 (15.0)	160 (18.0)	91 (21.0)	242 (18.3)	0.001
PA level, n (%)					0.742
Light	2061 (72.0)	628 (70.6)	317 (73.2)	938 (71.1)	
Moderate/Vigorous	802 (28.0)	261 (29.4)	116 (26.8)	381 (28.9)	
Smoking, n (%)					<0.001
Never	1999 (69.8)	592 (66.6)	278 (64.2)	829 (62.9)	
Former/Current	864 (30.2)	297 (33.4)	155 (35.8)	490 (37.1)	
Drinking, n (%)					0.650
Never	1823 (63.7)	547 (61.5)	272 (62.8)	843 (63.9)	
Current	1,040 (36.3)	342 (38.5)	161 (37.2)	476 (36.1)	
Education, n (%)					<0.001
Primary school and below	1,304 (45.5)	378 (42.5)	239 (55.2)	779 (59.1)	
Junior	1,046 (36.5)	242 (27.2)	110 (25.4)	315 (23.9)	
High school	397 (13.9)	240 (27.0)	77 (17.8)	202 (15.3)	
College and above	116 (4.1)	29 (3.3)	7 (1.6)	23 (1.7)	
Overweight/obesity, n (%)	1,380 (48.2)	418 (47.0)	184 (42.5)	542 (41.1)	0.078
Age (years)^a^	47.8 ± 0.7	51.8 ± 0.7	54.0 ± 0.5	55.5 ± 0.5	<0.001
Height (cm)^a^	159.93 ± 8.13	160.17 ± 8.19	159.44 ± 7.96	159.36 ± 8.57	0.831
Weight (kg)^a^	62.12 ± 10.98	61.75 ± 10.41	59.87 ± 10.38	60.11 ± 10.69	<0.001
BMI (kg/m2)^a^	24.24 ± 3.49	24.09 ± 3.66	23.53 ± 3.47	23.62 ± 3.60	<0.001

Abbreviations: CVDs, cardiovascular diseases; PA, physical activity; SD, standard deviation; BMI, body mass index.

^a^ Mean ± SD.

Table 2 shows the association between famine exposure in early life and risk of CVDs in adulthood. Compared with the unexposed group, fetal-exposed, infant-exposed and preschool-exposed groups had higher risk of CVDs in adulthood (*p* < 0.05), even after adjusting for gender (*p* < 0.05), and smoking status, drinking status, physical activity, the highest education attainment, BMI status, and famine severity (*p* < 0.05). Furthermore, sensitivity analysis also shows similar association (Supplementary Table S1).

**TABLE 2:** Associations between famine exposure and cardiovascular diseases risk, odds ratio (95% confidence interval) (China, 2011–2012).

Models	Unexposed group (n = 2,863)	Fetal exposed group (n = 889)	Infant exposed group (n = 433)	Preschool exposed group (n = 1,319)
Model 1				
OR (95% CI)	Ref	1.24 (1.02–1.52)	1.51 (1.17–1.94)	1.27 (1.07–1.51)
*p*-value	Ref	0.033	0.002	0.007
Model 2				
OR (95% CI)	Ref	1.26 (1.03–1.53)	1.55 (1.20–1.99)	1.30 (1.09–1.55)
*p*-value		0.026	0.001	0.003
Model 3				
OR (95% CI)	Ref	1.24 (1.01–1.52)	1.51 (1.17–1.95)	1.24 (1.04–1.48)
*p*-value		0.038	0.002	0.016

Abbreviations. OR, odds ratio; CI, confidence interval; Ref., reference. Model 1, unadjusted for any covariate. Model 2, adjusted for gender, Model 3 further adjusted for smoking status, drinking status, physical activity level, education level, and famine severity.

Associations between famine exposure and CVDs risk stratified by smoking status, drinking status, PA level, BMI status, and education level were presented in Table 3. The association between famine exposure and adulthood CVDs was not significantly modified by smoking status, drink behavior, and overweight/obesity status (*p*-values for interaction all >0.05). However, it seems that participants with above primary education level were more sensitive to early life famine exposure than that below primary education level (*P*_interaction_ = 0.043). In addition, sensitivity analysis also shows similar association (Supplementary Table S2).

**TABLE 3:** Associations between famine exposure and cardiovascular diseases risk, odds ratio (95% confidence interval) stratified by smoking status, drinking status, physical activity, body mass index, and education level (China, 2011–2012).

Groups	Fetal exposed group	Infant exposed group	Preschool exposed group	*p* for interaction
Severity				0.064
Less	0.93 (0.64–1.34)	1.78 (1.14–2.78)*	1.15 (0.84–1.59)	
Severe	1.46 (1.08–1.99)*	1.54 (1.05–2.24)*	1.33 (1.03–1.72)*	
Smoking status				0.906
Never	1.28 (1.00–1.64)*	1.46 (1.06–2.01)*	1.20 (0.96–1.50)	
Former/smoker	1.09 (0.76–1.56)	1.52 (0.99–2.33)	1.20 (0.89–1.62)	
Drinking status				0.725
Never	1.14 (0.89–1.46)	1.42 (1.03–1.94)*	1.18 (0.96–1.46)	
Former/drinker	1.36 (0.97–1.93)	1.66 (1.06–2.60)*	1.24 (0.90–1.71)	
PA level				0.180
Light	1.38 (1.09–1.76)**	1.36 (1.0–1.86)*	1.19 (0.96–1.47)	
Moderate/vigorous	0.93 (0.64–1.37)	1.83 (1.16–2.91)**	1.26 (0.92–1.73)	
BMI status				0.440
<24.0 kg/m^2^	1.03 (0.74–1.45)	1.42 (0.95–2.11)	1.14 (0.87–1.50)	
≥24.0 kg/m^2^	1.39 (1.00–1.93)*	1.98 (1.31–3.02)**	1.39 (1.03–1.86)**	
Education level				0.043
Primary and below	0.96 (0.68–1.33)	1.44 (0.99–2.09)	1.07 (0.82–1.38)	
Above primary	1.50 (1.07–2.09)**	1.99 (1.27–3.12)**	1.58 (1.14–2.18)**	

Abbreviations. PA, physical activity; BMI, body mass index. The Model adjusted for all the covariates (smoking status, drinking status, physical activity level, education level, and famine severity) except for the stratification covariate. *p < 0.05, **p < 0.01.

Table 4 shows the association between fetus and infancy famine exposure and CVDs risk in adulthood compared with age-matched control groups. Compared with the age-matched control group, fetal exposed group did not significantly increase adult CVDs risk (*p* > 0.05). However, infant exposed group still had a significantly higher risk of CVDs (OR = 1.52; 95% CI: 1.15, 2.01; *p* = 0.006). The association between famine exposure and adulthood CVDs was not significantly modified by smoking status, drink behavior, overweight/obesity status, and educational level (*p*-values for interaction all >0.05). Additionally, sensitivity analysis also shows similar association (Supplementary Table S3).

**TABLE 4:** Associations [odds ratio (95% confidence interval)] between fetus and infancy Chinese famine exposure and cardiovascular diseases risk compared with age-matched control groups (China, 2011–2012).

Stratified factors	Fetal exposed group	Infant exposed group	*p* for interaction
Total	1.11 (0.89–1.39)	1.52 (1.15–2.01)**	
Severity			0.164
Less	0.88 (0.62–1.25)	1.70 (1.10–2.61)*	
Severe	1.33 (0.99–1.78)	1.38 (0.96–1.99)	
Smoking status			0.634
Never	1.20 (0.91–1.58)	1.52 (1.06–2.18)*	
Former/smoker	0.94 (0.64–1.39)	1.48 (0.95–2.31)	
Drinking status			0.802
Never	1.06 (0.81–1.39)	1.49 (1.06–2.09)*	
Former/drinker	1.16 (0.78–1.72)	1.59 (0.98–2.59)	
PA level			0.064
Light	1.29 (0.99–1.68)*	1.35 (0.96–1.89)	
Moderate/vigorous	0.82 (0.54–1.25)	2.05 (1.25–3.36)**	
BMI status			0.465
<24.0 kg/m^2^	0.98 (0.71–1.36)	1.35 (0.92–1.98)	
≥24.0 kg/m^2^	1.25 (0.91–1.71)	1.79 (1.19–2.69)**	
Education level			0.427
Primary and below	0.93 (0.68–1.28)	1.40 (0.98–2.02)	
Above primary	1.30 (0.95–1.79)	1.73 (1.12–2.67)**	

Abbreviations: PA, physical activity; BMI, body mass index. The Model adjusted for all the covariates (smoking status, drinking status, physical activity level, education level, and famine severity) except for the stratification covariate.*p < 0.05,**p < 0.01.

## Discussion

We observed that infants exposed to severe famine significantly increased CVDs risk in adulthood, even compared to age-matched control. In addition, the association among subjects above primary education level appear to be stronger than that below primary education level. Indicating that early famine experienced may be responsible for the CVDs increasing in China, and a later ‘richer’ nutrition condition could exacerbate the effect.

Though the mechanisms that underlie the association between infancy famine exposure and adulthood CVDs risk yet to be elucidated, several potential mechanisms might contribute to the association. First, individuals who suffered from severe intrauterine malnutrition may increase the intake of a high-fat diet in adulthood while reducing the level of physical activity, as observed by a Dutch famine study [32]. Both high-fat foods and low physical activity are associated with the higher CVDs risk [33, 34]. However, we did not observe significant low PA among famine exposure groups (Table 1), suggesting low PA might not be that important in famine-CVDs association. Second, experience of famine during infanthood may alter the expression of the renin-angiotensin system (RAS), subsequently alter the renal vascular and tubular structures, and increase the risk of hypertension in adulthood, as suggested by animal studies [35, 36]. Third, the changed DNA methylation level of cardiovascular metabolic genes may play a key role in association between famine exposure in early life and adulthood CVDs risk. Our previous findings have showed that fetus famine exposure was associated with higher DNA methylation of *INSR* and *IGF2* genes, which were also linked with lipid indicators [18, 19].

In the current study, we observed that infants exposed to the famine increased 52% CVDs risk in adulthood. It was inconsistent with previous results from the Leningrad famine and the Dutch famine, which did not observe significant difference of CVDs prevalence between early-life famine exposed groups and unexposed group [21, 22, 37]. Despite that the Dutch famine found no increase in prevalence and mortality from cardiovascular disease after prenatal famine exposure [22, 37], they reported that adolescents who exposed to the Dutch famine had a significantly higher CHD risk in adulthood among women. However, children (0–9 years) exposed to the famine did not increase the risk of CHD in adulthood [38]. In addition, they did not observe significant association between famine exposure and stroke [39]. We speculated that severity and lasting time of famine exposure among the Leningrad famine, Dutch famine and China Great famine might contribute to the inconsistent association. The China Great famine affected about 600 million population, lasting for three years and lead to approximately 30 million premature deaths [40, 41]. However, the Leningrad famine only affected 2.9 million people (including 500,000 children), and 630,000 died from hunger-related causes, and the Dutch famine only lasted for 6 months [42].

The current study used the nationally representative data found that infancy exposed to the China Great famine was associated with CVDs risk in adulthood, and the association seems to be stronger in population above primary education level. We observed that subjects above primary education level had a significantly higher CVDs risk associated with infant famine exposure than that below primary education level (OR 1.44 vs. 1.99; *P*_interaction_ = 0.043). Higher education attainments is associated with a higher income and richer nutrition condition in later life. Thus, the poor intrauterine nutrition condition mismatch with the richer nutrition condition in later life might furthermore exacerbate the adverse effect of famine exposure in early life on CVDs in adulthood. Despite we did not observe the significant interaction between famine exposure and BMI status on CVDs risk, the association between early-life famine exposure and adult CVDs risk seems to be stronger in subjects with BMI ≥24.0 kg/m^2^ than that with BMI <24.0 kg/m^2^.

The present study used the large national epidemiological survey data observed that infancy famine exposure was associated with higher CVDs risk in adulthood, and the association seems to be stronger among population above primary education level. However, several limitations should be mentioned. Firstly, despite we adjusted for the effect of age gap between exposed group and unexposed group using the age-matched control group, we cannot separate completely the effect of age gap from famine exposure. Secondly, the selection bias is unavoidable. Severe famine exposure in early life could eliminate weaker participants and remained healthier participants, which may decrease the real effect of famine exposure. Thirdly, CVDs was defined based on self-reported information and had not been validated by a sub-sample of the population studied, which might suffer from recall errors and misclassification of CVDs. However, the non-differential misclassification is likely to lead to an underestimated association. Fourth, subjects who exposed to famine in fetal period may also experience actual exposure in infant period partly due to China Great famine lasting for three years. There was no good method to distinguish accurately whether they were fetal-exposed or infant-exposed to famine. However, the current study defined infant-exposed cohort born from January 1, 1958 to December 31, 1958, which insured almost all the participants in this group were exposed to famine in infanthood. Fifth, due to adjustments to the limiting factors of CVDs, such as maternal kidney function and serum uric acid, the study might still have confounding bias.

## Conclusion

Early-life exposure to the China great famine might elevated the risk of CVDs in adulthood. The famine-CVDs association might be modified by later higher education attainments.

## Data Availability

Publicly available datasets were analyzed in this study. This data can be found here: http://charls.pku.edu.cn/zh-CN/page/data/2011-charls-wave1.
