# Supplementary material for: Early-Life Exposure to the Chinese Great Famine and Later Cardiovascular Diseases
Source: Int J Public Health. 2021 Mar 9;66:603859. doi: 10.3389/ijph.2021.603859 (PMC8565276; doi:10.3389/ijph.2021.603859)
Supplement: Supplementary file 1 [file DataSheet1.PDF]

Supplemental materials

Early-life exposure to the Chinese Great Famine and later cardiovascular diseases

Zhenghe Wang, Yanhui Dong, Rongbin Xu, Xijie Wang, Yanhui Li, Zhiyong Zou

Table S1 Sensitivity analysis of associations between famine exposure and CVDs prevalence risk, odds ratio (95% confidence interval)

Models	Unexposed group (n=1227)	Fetal exposed group (n=889)	Infant exposed group (n=433)	Preschool exposed group (n=1319)
Model 1				
OR (95CI)	Ref	1.21(0.96-1.52)	1.46(1.11-1.93)	1.23(1.01-1.52)
P-value	Ref	0.113	0.008	0.048
Model 2				
OR (95%CI)	Ref	1.23(0.98-1.55)	1.52(1.15-2.01)	1.28(1.03-1.57)
P-value		0.081	0.003	0.023
Model 3				
OR (95%CI)	Ref	1.20(0.92-1.57)	1.65(1.20-2.26)	1.27(1.00-1.61)
P-value		0.186	0.02	0.053

Note: Abbreviations: *OR*, odds ratio; *CI*, confidence interval. Model 1, unadjusted for any covariate. Model 2, adjusted for gender, Model 3 further adjusted for smoking status, drinking status, physical activity level, education level, and famine severity.

Table S2 Sensitivity analysis of associations between famine exposure and CVDs prevalence risk, odds ratio (95% confidence interval) stratified by smoking status, drinking status, PA, BMI, and education level.

Groups	Fetal exposed group	Infant exposed group	Preschool exposed group	*P for interaction*
Smoking status				0.884
Never	1.22(0.88-1.70)	1.58(1.06-2.35)^{*025}	1.19(0.88-1.61)	
Former/smoker	1.10(0.68-1.80)	1.75(1.02-2.98)^{*041}	1.43(0.94-2.17)	
Drinking status				0.046
Never	1.05(0.79-1.39)	1.30(0.92-1.82)	1.10(0.86-1.41)	
Former/drinker	1.66(1.08-2.57)*	2.06(1.23-3.47)**	1.53(1.01-2.32)*	
PA level				0.139
Light	1.43(1.08-1.89)*	1.42(1.01-1.99)*	1.23(0.96-1.59)	
Moderate/vigorous	0.86(0.56-1.33)	1.71(1.03-2.83)*	1.17(0.80-1.71)	
BMI status				0.426
$<$24.0 kg/m^2	0.98(0.67-1.44)	1.38(0.89-2.13)	1.10(0.80-1.54)	
≥24.0 kg/m^2	1.45(0.99-2.14)	2.12(1.33-3.37)**	1.45(1.02-2.07)*	
Education level				0.373
Primary and below	0.97(0.67-1.41)	1.46(0.97-2.22)	1.09(0.80-1.48)	
Above Primary	1.50(1.01-2.24)*	1.99(1.21-3.28)**	1.57(1.07-2.32)*	

Note: Abbreviations: PA, physical activity; BMI, body mass index. The Model adjusted for all the covariates (gender, smoking status, drinking status, physical activity level, education level, and famine severity) except for the stratification covariate. * $P < 0.05$, ** $P < 0.01$.

Table S3 Sensitivity analysis of associations [odds ratio (95% confidence interval)] between fetus and infancy Chinese famine exposure and CVDs prevalence risk compared with age-matched control groups

Stratified factors	Fetal exposed group	Infant exposed group	*P for interaction*
Total	1.05(0.83-1.33)	1.45(1.09-1.93)*	
Smoking status			0.658
Never	1.13(0.85-1.52)	1.44(1.00-2.08)	
Former/smoker	0.88(0.58-1.32）	1.42(0.89-2.26)	
Drinking status			0.678
Never	1.00(0.75-1.32)	1.39(0.98-1.97)	
Former/drinker	1.12(0.73-1.70)	1.58(0.95-2.62)	
PA level			0.065
Light	1.21(0.92-1.61)	1.29(0.91-1.83)	
Moderate/vigorous	0.78(0.51-1.21)	1.97(1.18-3.28)**	
BMI status			0.538
<24.0 kg/m^2	0.93(0.66-1.30)	1.30(0.87-1.92)	
≥24.0 kg/m^2	1.20(0.86-1.67)	1.75(1.14-2.67) *	
Education level			0.511
Primary	0.93(0.67-1.29)	1.40(0.96-2.03)	
Above Primary	1.19(0.85-1.67)	1.58(1.01-2.47)*	

Note: Abbreviations: PA, physical activity; BMI, body mass index. The Model adjusted for all the covariates (smoking status, drinking status, physical activity level, education level, and famine severity) except for the stratification covariate. * $P < 0.05$, ** $P < 0.01$.
